# Effectiveness of low-dose theophylline for the management of biomass-associated COPD (LODOT-BCOPD): study protocol for a randomized controlled trial

**DOI:** 10.1186/s13063-021-05163-2

**Published:** 2021-03-16

**Authors:** Trishul Siddharthan, Suzanne L. Pollard, Peter Jackson, Nicole M. Robertson, Adaeze C. Wosu, Nihaal Rahman, Roma Padalkar, Isaac Sekitoleko, Esther Namazzi, Patricia Alupo, John R. Hurst, Robert Kalyesubula, David Dowdy, Robert Wise, Peter J. Barnes, William Checkley, Bruce Kirenga

**Affiliations:** 1 Division of Pulmonary and Critical Care, University of Miami, School of Medicine, Johns Hopkins University, 1951 NW 7th Ave, Suite 2308, Miami, FL 33136 USA; 2 Center for Global Non-Communicable Diseases, Johns Hopkins University, Baltimore, USA; 3 Division of Pulmonary and Critical Care, Virginia Commonwealth University, Richmond, USA; 4 Department of Epidemiology, Bloomberg School of Public Health, Johns Hopkins University, Baltimore, USA; 5 Makerere University Lung Institute, Makerere College of Health Sciences, Kampala, Uganda; 6 UCL Respiratory, University College London, London, UK; 7 Department of Physiology, Makerere College of Health Sciences, Kampala, Uganda; 8 African Community Center for Social Sustainability, Nakaseke, Uganda; 9 National Health and Lung Institute, Imperial College, London, UK

**Keywords:** Biomass, COPD, Theophylline

## Abstract

**Background:**

COPD is a leading cause of death globally, with the majority of morbidity and mortality occurring in low- and middle-income country (LMIC) settings. While tobacco-smoke exposure is the most important risk factor for COPD in high-income settings, household air pollution from biomass smoke combustion is a leading risk factor for COPD in LMICs. Despite the high burden of biomass smoke-related COPD, few studies have evaluated the efficacy of pharmacotherapy in this context. Currently recommended inhaler-based therapy for COPD is neither available nor affordable in most resource-limited settings. Low-dose theophylline is an oral, once-a-day therapy, long used in high-income countries (HICs), which has been proposed for the management of COPD in LMICs in the absence of inhaled steroids and/or bronchodilators. The Low-dose Theophylline for the Management of Biomass-Associated COPD (LODOT-BCOPD) trial investigates the clinical efficacy and cost-effectiveness of low-dose theophylline for the management of biomass-related COPD in a low-income setting.

**Methods:**

LODOT-BCOPD is a randomized, double-blind, placebo-controlled trial to test the efficacy of low-dose theophylline in improving respiratory symptoms in 110 participants with moderate to severe COPD in Central Uganda. The inclusion criteria are as follows: (1) age 40 to 80 years, (2) full-time resident of the study area, (3) daily biomass exposure, (4) post-bronchodilator FEV_1_/FVC below the 5th percentile of the Global Lung Initiative mixed ethnic reference population, and (5) GOLD Grade B-D COPD. Participants will be randomly assigned to receive once daily low-dose theophylline (200 mg ER, Unicontin-E) or placebo for 52 weeks. All participants will receive education about self-management of COPD and rescue salbutamol inhalers. We will measure health status using the St. George’s Respiratory Questionnaire (SGRQ) and quality of life using the EuroQol-5D (EQ-5D) at baseline and every 6 months. In addition, we will assess household air pollution levels, serum inflammatory biomarkers (fibrinogen, hs-CRP), and theophylline levels at baseline, 1 month, and 6 months. The primary outcome is change in SGRQ score at 12 months. Lastly, we will assess the cost-effectiveness of the intervention by calculating quality-adjusted life years (QALYs) from the EQ-5D.

**Trial registration:**

ClinicalTrials.gov NCT03984188. Registered on June 12, 2019

**Trial acronym:**

Low-dose Theophylline for the Management of Biomass-Associated COPD (LODOT-BCOPD)

## Introduction

Regular inhalation of toxins, such as tobacco smoke, can result in chronic obstructive pulmonary disease (COPD), a heterogeneous disease marked by largely irreversible airflow obstruction of the small airways, chronic bronchitis, and emphysema due to complex gene-environment interactions over the lifetime [1, 2]. COPD affects approximately 328 million people worldwide and is a leading cause of death globally [3]. The vast majority (~ 90%) of morbidity and mortality occurs in low- and middle-income country (LMIC) settings. Although tobacco smoke is a leading risk factor for COPD globally, a significant proportion (20–30%) of COPD occurs among never smokers [4, 5].

Household air pollution (HAP) from biomass combustion is an important risk factor in the development of COPD in many LMICs. Globally, nearly three billion people rely on biomass, which includes wood, dung, and agricultural crop waste or coal, for cooking and heating [6]. Individuals exposed to HAP in LMICs are 41% more likely to have COPD than those without the exposure [7]. Biomass-associated COPD has a distinct histopathology, phenotype and inflammatory profile when compared to tobacco-mediated COPD, suggesting a differential response to treatment and disease prognosis compared to tobacco-mediated disease [1, 8]. Despite the high global burden of biomass-associated disease, little is known about the effectiveness of pharmacotherapies for biomass-associated COPD; to date, no clinical trials have focused specifically on treatment of biomass-associated COPD [8].

Theophylline has been used in the treatment of chronic obstructive airway diseases, including COPD and asthma, and remains widely prescribed worldwide, largely due to its low expense [9]. In many high-income countries, the frequency of side effects and the drug’s narrow therapeutic index, together with wide availability of newer and more effective therapies, notably long-acting bronchodilators, have led to reduced usage for management of COPD. However, a number of studies have demonstrated that theophylline at lower doses (1–5 mg /L) results in improved respiratory symptoms via transcriptional downregulation of inflammatory genes [10–12]. Therapeutic monitoring is not necessary at such doses. Low-dose theophylline has been proposed as a treatment for biomass-associated COPD in LMICs, where inhaler-based therapy for COPD is usually unaffordable, not available or both [9, 13]. Although no trials have been designed to evaluate the cost-effectiveness of treatment for COPD in LMICs, economic modeling demonstrates that annual per-capita costs for managing COPD with inhaler-based therapy would amount to USD 13,000–14,000 per disability adjusted life year (DALY) averted, well above cost-effectiveness benchmarks [14]. Previous studies among individuals with tobacco-associated COPD have demonstrated low-dose theophylline monotherapy results in improved lung function (FEV_1_), reduced respiratory symptoms, and decreased the frequency and duration of exacerbations [15].

The main objective of the Low-dose Theophylline for the Management of Biomass-Associated COPD (LODOT-BCOPD) trial is to assess the clinical efficacy and cost-effectiveness of low-dose theophylline for the management of biomass-associated COPD in a low-income setting.

## Design and methods

### Study setting

Uganda is a low-income country located in East Africa with a total population of 45 million and a million, over 80% of whom live in rural areas. The study will be carried out in Nakaseke, Central Uganda, a district covering 43,167 households with an estimated population of 208,500. Nakaseke is located 14 km from the nearest highway and has been defined as rural by the Uganda Bureau of Statistics [16]. Most of the inhabitants (75%) are subsistence farmers, and over 60% of them live on less than 45,000 shillings ($12) per month [16]. The ratio of physicians and nurses per person are 1:25,000 and 1:5000, respectively, making Nakaseke one of the most under-resourced health districts in Uganda. We have previously conducted a population-based study in Nakaseke using spirometry to assess the prevalence of COPD [17].

### Study population

For this study, we will enroll adults with grade B-D COPD living in Nakaseke, previously identified in the GECo study [17, 18]. GECo was a population-based study which enrolled 3624 participants to validate case-finding instruments and assess the effectiveness of COPD self-management plan. The inclusion criteria are as follows: (1) age 40 to 80 years, (2) full-time resident of Nakaseke (lived in the area for > 3 years), (3) daily biomass exposure, (4) post-bronchodilator FEV_1_/FVC below the lower limit of normal of the Global Lung Initiative Mixed Ethnic reference population [19, 20], and (5) GOLD Grade B-D COPD [2]. Exclusion criteria include the following: (1) plans to relocate within 1 year; (2) uncontrolled hypertension; (3) pregnancy; (4) current use of chronic respiratory medications (long-acting bronchodilators (LABA), long-acting muscarinic agents (LAMA), and inhaled corticosteroids (ISC); (5) pulmonary tuberculosis; (6) > 10 pack year tobacco smoking history and/or active smoking; and (7) known intolerance or contraindication to theophylline. All participants will additionally be COVID-19 tested by nasopharyngeal RT-qPCR 72 h prior to study visit.

### Study design

**Fig. 1:**
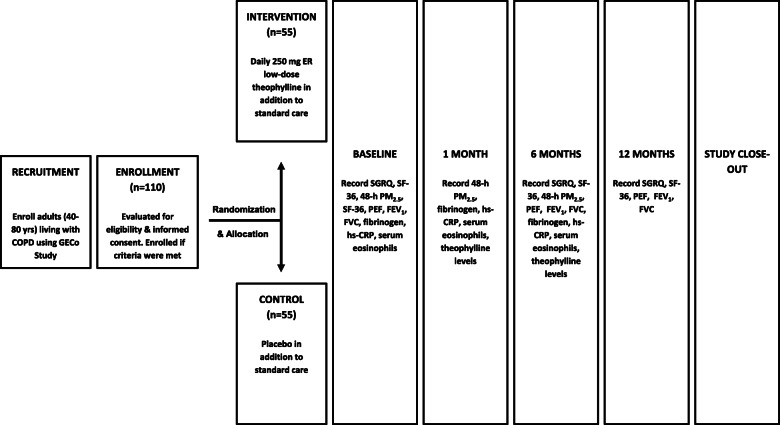
Recruitment diagram

We will enroll 110 adults in a randomized, double-blind, placebo-controlled trial to receive either daily 200 mg ER low-dose theophylline (“intervention”) or placebo (“control”) (Fig. 1). The primary outcome of the trial will be to assess the clinical efficacy of low-dose theophylline at 12 months as determined by differences in health states as measured by the St. George’s Respiratory Questionnaire (SGRQ). Secondary outcomes will include (a) differences in lung function decline and reversibility defined by ATS/ERS [21] and (b) differences in health-related quality of life measured by the Euro-Qol 5D (EQ-5D). We will additionally evaluate the biologic activity of low-dose theophylline in subjects by measuring circulating inflammatory biomarkers and assess whether theophylline decreased inflammation. Finally, we will estimate the incremental cost-effectiveness of the intervention.

Participants in the intervention group will be provided with a monthly supply of low-dose theophylline tablets in childproof bottles by trained research assistants. Both arms will additionally receive COPD specific education and salbutamol inhalers per standard care [22]. All participants will be followed monthly for a period of 12 months.

### Ethics

The trial protocol was approved by the Institutional Review Boards (IRB) at Johns Hopkins School of Medicine (IRB 209008), the University of Miami (IRB 20201523), The Makerere College of Health Sciences School of Medicine (REF 2020-093), and the Uganda National Council of Science and Technology (HS 2758). The trial was also registered with ClinicalTrials.gov (Identifier: NCT03984188) on June 12, 2019. Prior to any data collection, research staff will explain study purpose and procedures to participants, emphasizing that participation is completely voluntary and participants can choose to withdraw from the study at any time. Participants will also be provided with an information sheet. Written consent will be obtained from each participant for the present study and future ancillary studies, if applicable. In situations where a participant is unable to read or write, a thumbprint will be obtained, along with written signature from a witness. Access to identifiable individual-level data will be restricted to an independent study clinician and trial pharmacist. Protocol amendments will be approved by all regulatory parties prior to change in research activities.

### Training

All research personnel will receive human subjects training. Research assistants will be trained in electronic data capture and spirometry. Community health workers (CHWs) with experience in chronic respiratory diseases will provide COPD education [17]. CHWs have previously been educated on COPD pathophysiology, common treatments and their mechanism of action, as well as Ugandan guidelines for the diagnosis, management, and treatment of COPD, and familiarization with project goals.

### Recruitment, enrollment, and retention

Individuals with previously identified COPD will be recruited from the GECo study [17]. GECo was a population-based study which screened 3634 participants for COPD and enrolled subjects into a self-management trial. Trained research assistants will visit households to contact potential participants and invite them to the study. Before enrollment, study personnel will explain the goals of the study, what the study entails for the participant, and then ask if they are interested in participating. Those who agree to participate will be asked to complete detailed socioeconomic, medical history and exposure questionnaires and have spirometry performed for confirmatory testing.

### Randomization

Once a participant is determined to be eligible and agrees to enter the study, research assistants will block randomize them to each of the two groups using the automated randomization feature in REDCap (Vanderbilt University Medical Center, Nashville, TN, USA). Participants will be followed monthly for a 1-year period, and enrollment will be staggered over a 1-year period. Principal investigators, members of the data coordinating center, and participants will be blinded to treatment allocation. Unblinding will occur only at the discretion of the data and safety monitoring board (DSMB) or at time of final analysis.

### Study arms

Individuals randomized to the intervention arm will receive locally sourced low-dose theophylline (200 mg ER, Unicontin-E, Modi Mundi Pharma Pvt. Ltd.). Control randomized participants will receive methylcellulose placebo pills (Kampala Pharmaceutical Industries Ltd.) in identical packaging. The standard care for COPD as per WHO guidelines (salbutamol inhalers as needed) will be provided to all study participants, regardless of group assignment, by study clinicians. We will utilize standard dosing by ideal body weight (IBW). IBW is computed by using the Devine formulae: IBW_female_ = 45 + 0.9 (height in cm – 152) kg and IBW_male_ = 50 + 0.9 (height in cm – 152) kg [23]. A dose of theophylline ER 200 mg once daily (one placebo once daily) will be distributed to participants [24].

### Study outcomes

**Fig. 2:**
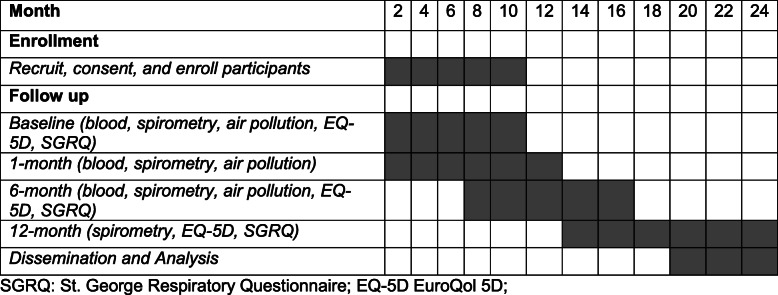
Schedule of enrollment, interventions and assessments

**Fig. 3:**
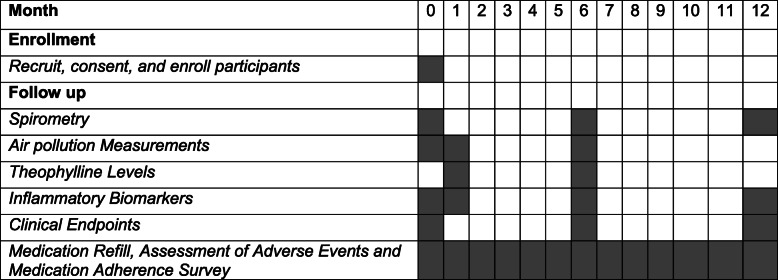
Schedule of enrollment, interventions, and assessments for participants

We will follow-up participants at 1 month, 6 months, and 12 months during face-to-face assessments in a clinical setting (Figs. 1, 2, and 3). In the event that a participant is unable to attend a scheduled follow-up assessment visit because of an acute illness (e.g., exacerbation of COPD) or other reasons, the visit can be postponed, to within 2 weeks of the scheduled assessment visit. Research assistants will additionally perform monthly visits to assess medication adherence, refill medications, and assess for adverse events.

### COPD questionnaires

The primary outcome of the trial will be a comparison of the change in SGRQ at 6 month increments from baseline to 12 months between the two arms [25]. The SGRQ measures impaired health and perceived well-being among individuals with chronic airway disease and offers many advantages for our study, namely (i) can be used to quantify changes in health following treatment, (ii) it is not limited to individuals with COPD, and (iii) it provides a standard metric that can be used for easy comparison across settings [25, 26]. We have previously validated the SGRQ in Luganda, the most widely spoken language in Nakaseke [27]. We will additionally administer the COPD Assessment Test (CAT) at 6-month increments. The CAT measures the impact of COPD (cough, sputum, dyspnea, chest tightness) on health status [28].

### Quality of life

The EuroQol-5D (EQ-5D) questionnaire will be administered at baseline and every 6 months through the trial period. The EQ-5D is a generic instrument for measuring health utility. It is based on a descriptive system that defines health in 5 dimensions: mobility, self-care, usual activities, pain/discomfort, and anxiety/depression [29]. Each dimension has 3 response categories corresponding to no problems, some problems, and extreme problems. The instrument is designed for self-completion, and respondents also rate their overall health on the day of the interview from 0 to 100 hash-marked, visual analog scale (EQ-VAS). Quality-adjusted life years (QALYs) will be derived using health utilities as estimated from the EQ-5D. The EQ-5D has been widely tested and used in both general populations and patient samples and has been locally validated in Uganda [29].

### Lung function

Spirometry will be conducted on all participants before and after bronchodilator therapy (400 mcg of salbutamol using a spacer) following standardized guidelines [19]. We will use the Easy on-PC handheld spirometer (ndd, Zurich, Switzerland), a device that has been validated and used in several large population-based studies [30, 31]. We will record post-bronchodilator PEF, FEV_1_, and FVC.

### Biomarkers

We will assess fibrinogen levels, a biomarker for all-cause mortality and exacerbations among those with COPD [32–34]. We will additionally measure serum hs-CRP and blood eosinophils. We will conduct blood draws at baseline, 6 months, and 12 months with the aim of assessing response to theophylline as well as identifying sub-groups which may have a differential response to therapy. We will process and store serum samples in Uganda at − 80 °C for future analysis. Samples will be destroyed at the request of participants.

### COPD exacerbations

We will utilize ATS/ERS guidelines regarding exacerbation definition and severity. A COPD exacerbation will be defined as a worsening of patient’s dyspnea, cough, or sputum beyond day-to-day variability [35]. Exacerbations will be treated with antibiotics and/or oral corticosteroids by study clinicians per standard protocol in both arms. A minimum of 2 weeks between consecutive exacerbations/hospitalizations will be used to consider events as separate in follow-up analysis [24].

### Health-care utilization

Data on participant health care utilization will be collected. We will additionally collect data on concomitant medications/studies, outpatient visits, and any-cause hospitalizations during the previous month.

### HAP measurements

We will measure personal PM_2.5_ concentrations using the UPAS (Access Sensor Technologies, Fort Collins, CO), a gravimetric and real-time sampler. We will additionally collect continuous PM_2.5_ data using the OPC-N3 (Alpha Sense, Essex, UK). Participants will be encouraged to wear the monitors continuously during the 48-h period and to keep close while sleeping. Black carbon content of each personal filter will be determined using a validated optical attenuation measure [36, 37].

### Medication adherence

We will measure adherence to prescribed medications at baseline and during follow-up visits. First, we will use the Adherence to Refills and Medications Scale-7 (ARMS-7) [38]. ARMS-7 will be administered at baseline and monthly during the follow-up period. The score is calculated by summing the scores for all items; lower scores indicate better adherence. Second, we will collect empty drug bottles and unused medication; compliance will be assessed by pill count [39]. Third, we will measure inhaler use by counters placed on salbutamol.

### Statistical analysis plan

The primary study outcome will be to assess whether the low-dose theophylline intervention results in improved mean self-reported respiratory symptoms (SGRQ) compared to standard care at 12 months. For repeated outcome measurements (e.g., SGRQ, CAT, PEF, FEV_1_, FVC, serum biomarkers), linear mixed effects models will be used to account for within-subject correlation. The main analysis will be by modified intention-to-treat (ITT) based on cases where the primary outcome is available and will therefore rely on an assumption that data is missing at random. We will describe the number (%) with missing primary outcome, look at reasons for missing the outcome, and consider characteristics of the patients excluded from the ITT analysis. Multiple imputation for the primary analysis will be used if the missing data exceeds 10% of randomized patients and as a secondary analysis regardless of the level of missingness.

Exposures (e.g., PM 2.5) at each follow-up will be aggregated to represent chronic exposure over the study period, as determined by the health outcomes. Analyses will be stratified to assess consistency across communities (rural and peri-urban) and be combined to obtain an overall risk estimate. In the combined analysis, we will adjust for community-level confounders.

We will examine repeated measurements of SGRQ by treatment group and carry out exploratory analyses to consider effects of the intervention over time. The SGRQ has previously been shown to have a standard deviation of 19.5 points in a similar population and a minimal clinically important difference of 4 points (a previous study involving low-dose theophylline resulted in a 7.8 point difference between intervention and control) [15]. A sample of 99 participants with COPD total will be needed to produce an 80% two-sided confidence interval that excludes a 4-point difference in SGRQ under the scenario of a 7.8 point difference in means [40]. We anticipate recruitment of 110 participants to account for attrition (55 per arm).

We will additionally conduct exploratory analysis to compare the exposure-response relationship between HAP and FEV_1_ between study arms to assess whether theophylline attenuates the association. For the exposure-response associations, analyses will be conducted within the intervention and the control groups separately, as well as in a combined analysis. Non-linear associations between exposure and health outcomes will be examined using generalized additive models and other spline-based approaches [41]. We will estimate whether theophylline modifies the effect of HAP on lung function and respiratory outcomes via a principal stratification approach [42–44].

For evaluation of cost effectiveness, we will utilize measurements of the EQ-5D at baseline and months 3 and 6 to convert scores into health utility estimates using validated conversion formulae [45]. The incremental number of QALYs gained, comparing intervention participants to controls, can then be calculated by measuring the longitudinal values of health utility over the intervention period in each arm. We will adapt existing costing surveys from the parent trial in Uganda to adopt a societal perspective and include costs to participants, specifically monitoring productivity losses and costs from illness, in addition to costs of the program and the costs to society. For purposes of the cost-effectiveness analysis, the effectiveness of the intervention will be estimated as the incremental number of QALYs gained, as estimated from change in EQ-5D.

Uncertainty in the inputs of the cost-effectiveness analysis will be explicitly incorporated into the cost-effectiveness analysis using probabilistic methods. Uncertainty in incremental costs and health benefits of each strategy will be presented using a scatter plot (the cost-effectiveness plane), and the probability of each implementation strategy being considered cost-effective for a range of thresholds will be presented using a cost-effectiveness acceptability curve, displaying the probability of the intervention being cost effective at various levels of willingness-to-pay.

There is no universal benchmark for cost-effectiveness. Therefore, we will benchmark the cost-effectiveness of the intervention against a range of established willingness-to-pay thresholds [46]. We will compare the ICER to the WHO standard of gross domestic product per capita per QALY gained [47]. We will additionally compare our ICER to published estimates of incremental cost-effectiveness for other similar health interventions in Uganda. We will lastly compare the total cost of the intervention to the average annual household income among participants [46].

### Data management and quality assurance

Questionnaire-based data will be collected using REDCap (REDCap, Vanderbilt University Medical Center, Nashville, TN, USA) on password-protected tablet computers (Galaxy Tab A 10.1, Samsung Electronics Co., Suwon, South Korea) by trained research assistants. To protect confidentiality, all participants will be assigned a unique identification code, which will allow data to be de-identified and stored without identifying information. Identifiable information will only be accessible to the independent study physician, trial pharmacist, and members of the DSMB. Due to the low risks associated with this intervention, there are no pre-specified stopping rules though we will conduct an interim analysis at 6 months. We will conduct monthly checks of the data to assess completeness and outliers (data manager, principal investigator).

### Adverse events

In previous trials, there were no significant differences between low-dose theophylline and placebo group. The most frequent drug-related adverse effects were stomach discomfort, headache, insomnia, and palpitations. We will collect data on safety and tolerability of theophylline, placebo, and salbutamol inhalers monthly, as well as provide contact information for study clinicians. We will maintain a data safety and monitoring board (DSMB) with reporting of all serious adverse events within 24 h by a study clinician. We will utilize health monitoring infrastructure of the parent trial to adjudicate adverse events. Health centers will be identified based on participants’ residence and patients will be referred and transported for health-related events. The DSMB will audit trial conduct and adverse events every 12 months. Provisions for ancillary and post-trial care will be provided through trial insurance. We will report all adverse events in follow-up publication.

### Role of funder

This study is funded by the National Institutes of Health (NHLBI/NIH). Peer review of the original grant application contributed to the final study design. A representative of the funder may attend DSMB meetings, though the funder otherwise has no role in the conduct or analyses of the study.

### Dissemination and data sharing

The study results will be submitted for publication in peer-review journals and presentation at international meetings. Authorship will be determined by International Committee of Medical Journal Editors guidelines. The results will be additionally provided to the Ugandan Ministry of Health to develop national guidelines. We will make limited, de-identified datasets available for reproduction of any published analysis.

## Discussion

This article discusses the rationale, methods and protocols for the LODOT-BCOPD randomized controlled trial in Uganda. The overall goal of the trial is to evaluate clinical efficacy and cost-effectiveness of low-dose theophylline treatment for biomass-associated COPD in a rural community in Uganda, in the absence of long-acting bronchodilators and/or inhaled corticosteroids.

This study will include a total of 110 patients randomized in a 1:1 ratio to low-dose theophylline or placebo. Data will be collected to evaluate health-status improvement with widely used and locally validated questionnaires. In addition, lung function and biomarker testing will provide objective data to compare the two groups. Finally, analysis will evaluate the cost-effectiveness for the therapy, which is paramount for a disease that disproportionately affects LMICs. This study will be conducted in Nakaseke, Uganda, by a local and multi-national research group with extensive experience conducting research in the region. This local experience will assist in navigating economic and cultural barriers to enrollment and allow community buy-in. This clinical trial builds upon previous research and collaborations in LMIC settings [17]. This previous research created well defined cohorts of patients with biomass COPD and has laid the groundwork for community partnerships that will facilitate recruitment and trust in the trial interventions. Furthermore, previous work has built capacity in the region and educated Ugandan nurses, physicians, and CHWs in research design and implementation.

This study has the potential to change the way we understand and treat biomass-associated COPD. Currently, there are no treatments that have been formally studied for biomass-associated COPD. Furthermore, in LMICs, where this disease is prevalent, standard COPD treatments are not widely available and unaffordable. This trial, if successful, could change the treatment strategy for millions of patients worldwide with this disorder and is scalable given the ease of administration and low cost.

### Trial status

Enrollment for LODOT-BCOPD started on February 23rd 2021. Estimated date of completion is March 1 2023. Protocol version 1.4, March 1, 2021.

## Data Availability

Not applicable.

## Abbreviations

LODOT-BCOPD: Low-dose Theophylline for the Management of Biomass-Associated COPD
COPD: Chronic obstructive pulmonary disease
LMICs: Low- and middle-income countries
HAP: Household air pollution
DALY: Disability-adjusted life year
FEV_1_: Forced expiratory volume
GECo: Global excellence in COPD outcomes
FVC: Forced vital capacity
ER: Extended release
IRB: Institutional Review Board
CHWs: Community health workers
IBW: Ideal body weight
SGRQ: St. George’s Respiratory Questionnaire
EQ-5D: EuroQol-5D
CAT: COPD Assessment Test
QALYs: Quality-adjusted life years
PEF: Peak expiratory flow
hs-CRP: High-sensitivity C-reactive protein
ATS: American Thoracic Society
ERS: European Respiratory Society
PM: Particulate matter
ARMS-7: Adherence to Refills and Medications Scale-7
ITT: Intention to treat
